# Nurse-led decision coaching by specialized nurses for healthy *BRCA1/2* gene mutation carriers - adaptation and pilot testing of a curriculum for nurses: a qualitative study

**DOI:** 10.1186/s12912-022-00810-8

**Published:** 2022-02-10

**Authors:** Birte Berger-Höger, Frank Vitinius, Hannah Fischer, Karolina Beifus, Juliane Köberlein-Neu, Anna Isselhard, Maren Töpper, Regina Wiedemann, Kerstin Rhiem, Rita Schmutzler, Stephanie Stock, Anke Steckelberg

**Affiliations:** 1 Institute for Health and Nursing Science, Faculty of Medicine Martin Luther University Halle-Wittenberg, Halle (Saale), Germany; 2 Department of Psychosomatics and Psychotherapy, Faculty of Medicine, University Hospital Cologne, University of Cologne, Cologne, Germany; 3 Center for Health Economics and Health Services Research, Schumpeter School of Business and Economics, University of Wuppertal, Wuppertal, Germany; 4 Institute of Health Economics and Clinical Epidemiology, University Hospital of Cologne, Cologne, Germany; 5 Center for Familial Breast and Ovarian Cancer, Center for Integrated Oncology (CIO), University of Cologne, Faculty of Medicine and University Hospital Cologne, Cologne, Germany

**Keywords:** Decision making, Shared; decision coaching; oncology nursing, Breast care nursing, BRCA1/2, Curriculum development, Decision support techniques, Nursing education

## Abstract

**Background:**

Women with *BRCA1/2* mutations are at high risk to develop breast and ovarian cancer. To support these women to participate in shared decision-making, structured nurse-led decision coaching combined with an evidence-based decision aid may be employed.

In preparation of the interprofessional randomized controlled trial to evaluate a decision coaching program to support preventive decisions of healthy female BRCA 1/2 gene mutation carriers (EDCP-BRCA), we adapted and piloted an existing training program for specialized nurses and included elements from an existing physician communication training.

**Methods:**

The training was adapted according to the six-step-approach for medical curriculum development. The educational design is based on experience- and problem-based learning.

Subsequently, we conducted a qualitative pilot study. Nurses were recruited from six German centers for familial breast and ovarian cancer. The acceptability and feasibility were assessed by structured class observations, field notes and participants’ feedback. Data were analyzed using qualitative content analysis. The training was revised according to the results.

Due to the COVID-19 pandemic, the patient intervention was adapted as a virtual coaching and a brief additional training for nurses was added.

**Results:**

The training consists of two modules (2 + 1 day) that teach competences in evidence-based medicine and patient information, (risk) communication and decision coaching. One pilot test was conducted with six nurses of which three were specialized and experienced in patient counselling. A final set of eight main categories was derived from the data: framework conditions; interaction; schedule, transparency of goals, content, methods, materials and practical relevance and feasibility. Overall, the training was feasible and comprehensible. Decision coaching materials were awkward to handle and decision coaching role plays were set too short. Therefore, materials will be sent out in advance and the training was extended.

**Conclusions:**

Specialized nurses are rarely available and nurse-led counselling is not routinely implemented in the centers of familial breast and ovarian cancer. However, training of less qualified nurses seems feasible. Decision coaching in a virtual format seems to be a promising approach. Further research is needed to evaluate its feasibility, acceptability and effectiveness.

**Trial registration:**

The main trial is registered under DRKS-ID: DRKS00015527.

**Supplementary Information:**

The online version contains supplementary material available at 10.1186/s12912-022-00810-8.

## Background

Healthy women with breast cancer susceptibility gene 1/2 (*BRCA1/2* gene) mutations are at high risk to develop breast and ovarian cancer. Women with *BRCA1/2* gene mutations have a cumulative risk of about 70% up to the age of 80 for breast cancer and about 44% (*BRCA1*) and 17% (*BRCA2*) for ovarian cancer [1]. The lifetime risk of the general population is about 13 and 1% for breast and ovarian cancer respectively.

To deal with these risks, healthy mutation carriers can decide between different options. These options comprise an intensified breast cancer-screening regimen, prophylactic surgeries, such as bilateral mastectomy and/or salpingo-oophorectomy, or to take no action at the moment. All options differ in their benefit-harm profiles [2–7].

Women’s decisions are influenced, among other factors, by their level of anxiety, their desire to have children, their family history and experiences with cancer, their body image and (in some cases) attitudes as well as behavior of health professionals [8–10]. Recent research has shown that despite guideline-based genetic counseling including risk communication, in many cases women overestimate their 10-year-risk of breast and ovarian cancer [11].

Decision support is an essential need of women facing these complex decisions [12, 13]. Previous research indicates that women want to participate in decision-making and need decision support [14, 15]. Accordingly, the German patients’ rights act, the National Cancer Plan and the German medical treatment guideline for breast cancer confirm the patients’ rights of participation and informed choices based on evidence-based health information [16, 17]. Informed choice means that women are enabled to make their own choices based on adequate knowledge about all existing options and in congruence with their individual preferences [18].

One way to facilitate informed shared decision-making in an interprofessional team is decision coaching by trained healthcare professionals combined with evidence-based decision aids [19]. Decision coaching is a non-directive decision support [20]. Trained healthcare professionals provide decision coaching to patients aiming at the development of patient’s skills in 1) understanding and thinking about the options, 2) preparing for discussing their decisions in a consultation with his or her health professional, and 3) implementing the chosen option [19, 21]. Systematic reviews on shared decision-making interventions have shown that decision coaching is important to enhance patient autonomy and empowerment [19, 21]. Comparing decision coaching paired with a decision aid and usual care, there is low certainty evidence that decision coaching improves patients’ understanding of their care. It might improve patient participation in decision-making, enhance informed decisions, reduce costs, and led to intervention-specific positive outcomes such as quality of life particularly when nurses offer the coaching [20–23]. However, the evidence is uncertain [20].

Decision aids provide evidence-based information in a comprehensive, comprehensible, transparent and neutral manner [24]. They explicitly state the decision that needs to be made, provide evidence-based information about the condition, present the options including probabilities of benefits and harms that might occur and the scientific uncertainties underlying the evidence.

The Centers of the German Consortium for Familial Breast and Ovarian Cancer are committed to the principles of non-directive counselling and the shared decision-making concept in standard operating procedures, thereby ensuring an adequate risk communication about the risk of developing disease and the risk reduction by preventive surgeries [25, 26]. A detailed description of usual care can be found in the study protocol [27] and will be provided in the scope of the accompanying process evaluation.

The EDCP-BRCA study (DRKS-ID: DRKS00015527) (German acronym meaning evaluation of a decision coaching program to support preventive decisions of healthy female BRCA 1/2 gene mutation carriers) evaluates a complex intervention for healthy women with *BRCA1/2* gene mutations facing a decision on preventive options compared with optimized care [27].

The intervention (optimized care plus decision-coaching) is built on a training for specialized nurses providing decision coaching for women with ductal carcinoma in situ [28, 29] and comprises a nurse-led decision coaching in order to prepare medical consultations combined with an evidence-based decision aid [30].

The aim of this study was to adapt, complement and pilot an existing training program [29] for specialized breast care nurses and oncology nurses and included elements from an existing physician communication training. This nurse training aims to impart competences in decision coaching and evidence-based patient information.

## Methods

### Adaptation of the curriculum

The original curriculum [29] was adapted and piloted according to the Medical Research Council’s guidance for developing and evaluating complex interventions with focus on acceptability and feasibility (phase I and II) [31]. The reporting follows the revised Criteria for Reporting the Development and Evaluation of Complex Interventions in healthcare (CReDECI 2) [32], and the COnsolidated criteria for REporting Qualitative research (COREQ) (see Additional file 1) [33].

We adapted the training program following Kern’s six-step approach for curriculum development for medical education [34]:

#### Step 1: problem identification and general needs assessment

As outlined above, women with *BRCA1/2* gene mutations should be enabled to participate in treatment decision-making. Decision-making in this field is complex. Due to the uncertainty related to the gene mutation, women feel anxious and they need emotional and decision support beyond medical information [14].

#### Step 2: targeted needs assessment

Specialized nurses like oncology or breast care nurses who are already implemented into the care of breast cancer patients are suited for the role of decision coaches in centers for familial breast and ovarian cancer. Whereas internationally a master’s degree as advanced nurse practitioner has been established [35], most specialized nurses in Germany have done a one or two-year training course as a breast care or oncology nurse [36]. Specialized nurses are particularly suited for this new role as they are already mediators to explain medical information to patients [37] and patients’ advocates in oncological settings [38]. Currently, most specialized nurses in Germany are not educated in evidence-based health communication and decision coaching [39].

#### Step 3: goals and objectives

The teaching goals were adapted to the special needs in the field of decision coaching for women with *BRCA1/2* gene mutations. Learning goals of the existing curriculum for decision coaching [29] were adapted and complemented. Essential contents of an existing communication skills training program for physicians were adapted and integrated into the decision coaching training [40]. Goals and objectives are broadly defined in Table 1.

**Table 1:** Teaching goals and objectives

**Module 1: Basics of evidence-based decision making and evidence-based patient information**		
**Goals**	**Content**	**Educational strategies**
Nurses critically reflect the model of informed choice regarding their personal and work experiences.	• Informed choice	• Patient narrative: think, pair, share-method
Nurses describe and compare the different patient participation models paternalism, shared decision-making and the autonomous decision model for medical decision making.	• Models of medical decision making and patient participation: Paternalism, shared decision-making and autonomous decision model	• Lecture
Nurses acquire essential communication skills for decision coaching such as teach back and dealing with emotional situations.	• Dialogue techniques • Attentive listening during pauses of dialogue • Confidence building measures • Setting of communication • Dealing with emotions • Summarizing adequately • Teach back	• Lecture • Role plays (experienced learning)
Nurses deal with risks for their psychosocial strain in their professional field and the prevention of burn out.	• Psycho hygiene • Prevention of burn out	• Lecture • Exercise
Nurses identify the necessity of randomized controlled trials to proof the efficacy of an intervention and explain quality criteria of randomized controlled trials.	• Randomized controlled trials (example efficacy of vitamin D intake for cancer prevention) • Risk of bias and its prevention	• Group work and lecture
Nurses describe evidence-based medical guidelines as an implementation tool of evidence-based knowledge for practitioners using the example of the German breast cancer guideline (S3).	• Guideline • Grades of recommendation • Level of evidence	• Lecture
Nurses appraise commonly available patient information material with the criteria of evidence-based health information and their appropriateness to support informed decision making.	• Criteria of evidence-based health information	• Lecture, exercise, discussion
Nurses communicate the probabilities of benefits and harms for preventive options as well as predictive values in a comprehensible manner for patients according to the criteria of evidence-based health information.	• Relative risk • Absolute risk • Relative risk reduction • Positive and negative predictive values (example magnetic resonance imaging) • Uncertainty underlying the evidence	• Lecture, exercises, role plays
**Module 2: Shared decision-making and decision coaching**		
**Goals**	**Content**	**Educational strategies**
Nurses elucidate the distinct steps of decision coaching.	• Exploration of decision needs such as knowledge gaps, unclear values, lacking support or uncertainty • Provision of support such as health information, value clarification, enhancement of support from others and resources	• Lecture
Nurses conduct a simulated decision coaching using prompt cards, the decision guidance, the fact sheets and the decision aid and give feedback to each other.	• Decision coaching	• Role play with peers and simulated patients
Nurses take part in gene diagnostic boards and patient physician consultations and emphasize their role in the inter-professional team.	• Understanding of gene diagnostic test results and their interpretation • Overview of the center’s procedures and the decision-making process. • Gain an insight into mutation carriers’ motives to opt for preventive measure or to do not and women’s different coping strategies. • Nurse’s role in the inter-professional team	• Initial training plan; discussion

#### Step 4: educational strategies

Educational strategies were adopted from the original training. The educational design was guided by the theory of planned behavior and is based on experience- and problem-based learning [29]. We adapted the examples used in the training according to the topic. The training program consisted of two modules (Table 1). A folder containing the training materials was provided for every nurse.

The *first module* of the training program was planned as a two-and-a-half day in-class training that aimed to impart competences in the basics of evidence-based medical decision-making, the evidence of preventive options for healthy women with a BRCA1/2 gene mutation, the critical appraisal of patient information material based on the criteria of evidence-based health information [24] as well as risk and essential communication skills.

In order to enhance nurses’ communication skills, parts of an existing communication skills training for physicians were adapted for nurses [40]. Contents were similar to other communication skills trainings for healthcare professionals in the area of oncology and genetic counselling [41–44] (Table 1) and complemented e.g. with a theoretical input and exercises about mental hygiene.

In the *second module*, lasting one day, participants acquired decision coaching skills comprising the assessment of decisional needs and providing decision support, e.g. value clarification or the provision of health information according to the Ottawa Decision Support Framework [12]. The decision coaching is based on an evidence-based decision aid [30] comprising information on all available preventive strategies and, in cases where women tend to bilateral mastectomy, the opportunities of breast reconstruction. During the decision coaching, a decision guide targeted at the decisions for healthy women with *BRCA1/2* gene mutations is offered in order to structure and document the decision process. Additionally, nurses can use fact sheets displaying essential information of the decision aid. Prompt cards providing key phrases for each step of the decision coaching can be used in preparation to or during coaching by nurses. The results of the decision coaching discussions are summarized in a document sheet by the nurses and handed over to the physicians to encourage interprofessional information sharing.

To provide non-directive guidance and decision support, nurses were asked to reflect on their own experiences, attitudes, values, and preferences regarding this decision-making entity. Therefore, we chose experience-oriented and problem-based learning.

To impart decision coaching skills, role plays with peers and simulated patients were conducted and peer feedback as well as a tutor’s feedback was given to nurses. In preparation of the role plays, the actor patient was instructed using three case vignettes that were developed beforehand by researchers (HF, FV, BBH, AS), clinical experts (KR, RW) and in consultation with a self-help group member of the German BRCA-network.

In addition, an initial training plan for the nurses’ new working field has been released to nurses and directors at all centers for familial breast and ovarian cancer participating in the study. It comprises work shadowing of at least ten medical consultations in which genetic test results are disclosed to women and preventive strategies are discussed as well as the participation in at least one gene diagnostic board. The training materials were reviewed by experts in the field and patient representatives.

#### Step 5: implementation

This step corresponds to the pilot testing of the training program with the target group (see Section Piloting and feasibility of the training program).

#### Step 6: evaluation and feedback

We revised the training program according to the results of this qualitative pilot study before it was implemented for the training of specialized nurses in preparation of the randomized controlled trial in the EDCP-BRCA project.

### Piloting and feasibility of the training program

We conducted a qualitative pilot study with focus on acceptability and feasibility of the training program.

#### Setting and sample

The directors of the six centers for familial breast and ovarian cancer participating in the main study were contacted to recruit specialized nurses for the pilot testing. All centers belong to the German Consortium for Familial Breast and Ovarian Cancer, are part of university hospitals and spread throughout Germany [26]. All clinics perform genetic testing and are characterized by a large number of cases. Specialized nurses should have completed a one- or two-year training as breast care or oncology nurse. Participation was voluntary, counted as working time and travel costs were funded by the study center. In advance, some nurses were offered the opportunity of taking over this new role in the main trial, if they wanted to. The decision coaching training program was offered at the University of Cologne. The teaching program was designed for a maximum of eight nurses per course.

#### Data collection and procedure

Specialized nurses participated voluntarily in the training program. Written informed consent was obtained from every nurse at the beginning of the training. Training was carried out by AS, BBH, KR, HF and FV. Data collection was carried out during the training sessions by KB, JKN, BBH and AS, who gave a brief introduction to their research at the beginning of the training.

Baseline characteristics of the participants including sex, age, work experience as a nurse and in oncology, current working field and further trainings were assessed using a short, standardized questionnaire (see Table 2).

**Table 2:** Baseline characteristics of participants

Nurses	
Total, *n*	6
Women, *n*	6/6
Age, years, mean (range)	51 (35–61)
Practice in nursing, years, mean (range)	26 (10–43)
Work experience in oncology, years, mean (range)	8 (0–14)
**Working field**	
Center for familial breast and ovarian cancer, n	1/6
Breast care center, n	2/6
Other, n	2/6
Released from regular duties on the ward (100%), n	1/6
**Further trainings**	
Training as breast care nurse	3/6
Training as head nurse	2/6
Study nurse	4/6
Hygiene specialist	1/6
Mamma care trainer	2/6
No further training	1/6

Feasibility and acceptability of the training program were investigated from the perspectives of participants, trainers and observers. *Acceptability* was defined as the acceptance of teaching methods and the relevance of contents in consideration of their practical needs. *Feasibility* was defined as the practicability of the training program and its contents. At classroom level, we further focused on the following aspects: *comprehensibility* and *usability* of the learning and teaching materials and contents, structure of the training, scheduling and target group orientation. *Potential application barriers* and *participants’ motivation* were assessed with focus on practice implementation.

Open structured class observations were carried out by at least one non-participating observer of the accompanying process evaluation research team (JKN, KB) taking field notes. Foci of observations were the participants’ reactions to the teaching methods and materials, their comprehensibility, scheduling and the interaction between participants and trainers. The trainers (BBH, AS) also took fields notes, which were collated with the observers’ perception and discussed with observers afterwards in order to ensure a mutual understanding of the meaning. Work products such as notes on the white boards were documented by photographs.

At the end of each day, feasibility and acceptability were explored by the trainers (BBH, AS) from the individual participants’ perspective in flashlight-feedbacks. The feedbacks were recorded handwritten by trainers and observers. The notes and findings were not returned to the participants for comment and/or correction.

#### Data analysis

Analysis of the baseline characteristics were descriptive. The documentations (observers’ protocols and trainers’ field notes as well as the handwritten records of the participants’ feedbacks) were analyzed using qualitative content analysis according to Mayring [45] in order to summarize and structure the results according to the predefined aspects of feasibility and acceptability of the training. The different perspectives (observer, teacher and participants) were triangulated [46]. BBH, who is experienced in pilot testing of training curricula and qualitative analysis, coded the documents applying a coding guideline [Additional file 2] and using the software MAXQDA® [47]. Initially, a category system deductively derived from the research questions was applied to the data material. During the coding process, categories were adapted, and subcategories were inductively derived from the data. Afterwards, the coding results were discussed with another researcher (AS). The revision was guided by the results and discussions of the trainer team (FV, HF, AS and BBH). Results are reported according to the main categories (for the entire coding scheme see Additional file 2).

### Adaptation of the decision coaching intervention into a virtual format and pilot testing

Due to the lockdown and contact restrictions as a result of the COVID-19 pandemic, the decision coaching intervention was converted into a virtual decision-coaching and tested with one nurse from a participating study center. Therefore, the decision guidance was converted into an editable virtual document and the fact sheets were transformed into a virtual presentation so that nurses could easily navigate through it. The decision coaching should be conducted via the web-conference software Webex (CISCO). Instructions for the web-conference software and the adapted coaching material in preparation for a 90-min online-training was provided for the nurses. During the training, nurses could familiarize themselves with the software, ask questions, receive instructions for trouble shooting and one decision-coaching consultation was simulated using the virtual materials.

The training was pilot tested with one nurse. At the end of the training, she was interviewed with focus on acceptance and feasibility of the material and training. Furthermore, she was asked about her expectation to adopt this new intervention into her current decision coaching practice. The training and interview were recorded and transcribed verbatim. Field notes were taken by the trainer (BBH) and one observer (AS). Qualitative content analysis was conducted according to Mayring with focus on acceptability and feasibility [45].

## Results

The pilot test comprised six nurses and was conducted at the University of Cologne in July 2019 and October 2019. The additional pilot test of the virtual format was conducted in June 2020. Participating nurses were from four centers of the German consortium for familial breast and ovarian cancer. All nurses completed the training program. The average experience in nursing was 26 years (range: 10–43) (Table 2). Three had training as breast care nurses; none had training as an oncology nurse. Four out of the six nurses had experience in oncology with an average time of eight years (range: 0–14). Four nurses were currently working as study nurses. Table 2 shows the baseline characteristics.

A final set of main categories was derived from the data via qualitative content analysis: 1. Framework conditions of the training, 2. Interaction, 3. Schedule, 4. Transparency of teaching and learning goals, 5. Content, 6. Methods, 7. Materials and 8. Practical relevance and feasibility.

### Framework conditions and organizational aspects of the training

Participants and observers appreciated the positive training atmosphere. However, there were several organizational problems that hindered the training. The schedule for the second module had not been released to all participants in time and most of the nurses were not aware of their roles in terms of their upcoming tasks in the study. Most nurses were waiting for the initiation of the main trial to learn about their roles as study nurse. Their focus relied on administrative issues e. g. contracts with the main study center, the ethical approval of the study, the responsibilities within the trial and data protection issues whereas the focus on the decision coaching contents was minimized meanwhile.

Contrary to the previous planning, the decision aid had not been sent to the participants in preparation of the training, which negatively impacted the training.

### Interaction

All teachers were aware of their tasks and roles. Participants experienced the selection of methods as various and dynamic. The atmosphere between learners was open. By reflecting on own experiences with complex decisions, one nurse shared a very personal experience and started her narrative saying:

“*We are all so open here, so let me tell you something*...” (Nurse 1, module 1).

The participants wished to stay in touch to share experiences in the aftermath of the training. Upcoming questions were answered adequately by peers or teachers. The learners perceived the learning space as protected in terms of the role plays with peers and the simulated patient.

### Schedule

The teachers adapted the schedule (Table 3). The nurses’ divergent expectations with respect to the main objectives of the training, their insufficient previous training and their poor experiences in this working field resulted in extended discussions and requests.

### Transparency of teaching and learning goals

For large parts of the first module learners were not aware of the training’s main target.

„*I as a non-expert observer missed a brief description of the nurses’ task profile as a decision coach and of the required skills (e. g. expertise in terms of gene mutation, test accuracy, communication skills* etc.*) Furthermore, no general learning goals for the entire training were derived*.” (Observer protocol, module 1).

Learners were not able to place parts of the content into the overall context of training e. g. dealing with emotions or the input about *BRCA1/2* gene mutations.

*“[It was] positive to reflect one’s own communication again. He [the teacher] only talked about it, but the knot was not undone. It was too emotional”* (Nurse 3, module 1).

On the second day, teachers caught up on the learning goals. However, nurses often took on the perspective of a study nurse and less frequently that of a soon-to-be decision coach.

*„We need to know this [organizational aspects], so that the study can keep going* “(Nurse 3, module 1).

One nurse stated that she was unsettled by the lack of clarity of goals and wondered if she would be adequate for the task:

“*Presumably, theoretical basics are important. Studies and so on are important (and their appraisal), what could this look like. Everyone wondered if they would be able to work in it, would they be able to cope with it.*” (Nurse 2, module 1).

Some exercises were perceived as too extensive such as the impartation of competences in evidence-based medicine and test accuracy. At the end of the entire training, nurses reported that in hindsight, most of the content made sense to them and were relevant for their new task.

### Contents

In general, participants appraised the training as sophisticated. Most of the contents were comprehensible, even though they felt that some were challenging e. g. the calculation of test accuracy. In the first part of the training, most questions were related to study procedures. At the end of the training, participants indicated that they had identified knowledge gaps which they wanted to work on. In addition, they wanted to work out their own structure for the decision coaching consultations.

From the observer’s perspective, enough time had been spent on reflection. However, one participant showed a contradictory attitude towards the intake of vitamin D. Despite its proven non-effectiveness, she was convinced that vitamin D would have a protective effect regarding breast cancer. On the other side, one participant criticized that the breast self-examination has been recommended in the decision aid despite its non-effectiveness.

In the first module, participants desired more information to deal with relatives who turn out to be obstructive in the decision-making process. One participant asked for more information about decision theories.

Since most participants did not meet the criteria of the target group, their communication and counseling skills as well as their experiences with the needs and concerns of women with *BRCA1/2* mutations were below expectations. Participants desired more exercises and practical implications within the first module. The participants were partly familiar with communication skills, such as teach back. They described its theoretical background as helpful regarding future coaching sessions.

“*Misunderstanding of the training goal (expected initiation) and participants criticized a lacking initiation*

- *clarification by the project coordinators*
- *requested study protocol to better assign training contents to the study*” (Observer protocol, module 1)

### Methods

The participants appreciated the instructional design as attractive and various:

*“Various methods, vivid.”* (Nurse 2, module 1).

Two participants disliked the role play but evaluated them as worthwhile. In module two, the time frame for nurses to familiarize themselves with the decision coaching material (decision aid, decision guidance, fact sheets) was set too short.

Furthermore, the 15 min allotted to each participant to practice decision coaching with a simulated patient was too short. All participants struggled to find a dialogue structure according to the decision coaching concept and to use the decision coaching material as intended. After several attempts of the nurses and qualified feedbacks from teachers, the expected training curve increased only slowly.

The offer to make personal notes in the decision guidance and to use it like a cheat sheet was only picked up by a few nurses.

### Materials

The participants received a training folder at the beginning of the training. Participants had difficulties finding the work sheets in the training folder, as they were filed behind the presentation slides and not in-between. The presentation slides containing the input about the *BRCA1/2* gene mutations were in English and no script was handed out to the participants, some slides were skipped.

Revision needs for the coaching materials in terms of structure and content were identified.

*“The active training with the simulated patients showed that participants were not familiar with materials (decision aid and decision guidance). Feedback: “You did not feel confident”* (Observer protocol, module 2).

### Practical relevance and feasibility

Participants appreciated the elucidation of challenging emotional situations. However, for some participants, the relevance did not become clear before the second module. Some wished to share their experiences after the training.

*“What a pity, that no meetings with the other study centers are scheduled to share experiences within the study. It would be great to share experiences and receive further training, especially in the case of establishing such a new intervention.”* (Nurse 4, module 2).

### Revision

The intervention has been adapted according to the results (see Table 3).

**Table 3:** Results of analysis and revision process

Identified needs for revision	Revision conducted
Participants were not familiar and confident in dealing with the coaching material.	The decision coaching material (decision aid, decision guidance, prompt cards and fact sheets) combined with a working sheet will be sent to the participants in preparation of the training.
Participants were not able to place some of the contents in the overall context of training e.g. dealing with emotions or the input to BRCA1/2 gene mutations.	The schedule of contents was revised.
The time frame given to each participant to practice decision coaching with a simulated patient was too short.	The time frame was expanded for each participant.
The time limit for some exercises and presentations were overstepped.	Work sheets were shortened, or they were revised to be done collaboratively. The presentation slides for risk communication, evidence-based health information and test accuracy were optimized in length and structure.
At the beginning participants were not clear about main goals of the training.	Main targets and aims of the training were presented at the beginning of the training
The input phase of BRCA1/2 were not standardized and slides were in English.	The input was skipped, the decision aid was sent out in preparation. In case of questions that require special expertise, questions are forwarded to an expert and the feedback is given to learners.
Calculation of test accuracy was too complex.	The calculation was reduced to the predictive values.
Work sheets were not easy to find in the training folder.	Work sheets were replaced in the training folder.
Decision coaching materials (decision guidance and fact sheets) were not easy to handle.	Decision guidance was divided into chapters (decision about preventive options for breast cancer and decision about preventive strategies for ovarian cancer). Fact sheets were adapted and reduced to a maximum of two sheets for each preventive option. For each option the sheets were bound.

An adequate introduction into the main objectives of the training was placed at the beginning of the training. After the pilot training, four nurses decided to take over the task of the decision coach within the main study. Therefore, a one-day refresher training was provided with revised coaching materials to ensure adequate decision coaching skills of all nurses three months after the previous training.

### Results of pilot testing of the training for virtual decision coaching

#### Acceptability

The nurse felt well prepared for the new concept and expected that it would be well accepted by women, irrespective of the pandemic. Especially women from rural areas would prefer this mode of delivery.

*“Apart from that, it was really helpful to simply get a feeling for it and also how to use the different tools “*(Nurse).

#### Feasibility of the virtual decision coaching intervention

The material (decision guidance and fact sheets) as well as the web conference software were appropriate and no revision was needed. The nurse desired more training with simulated patients and felt uncertain with regard to her technical skills.

“*Well, that I, that I would feel anxious that something goes wrong with the technics, that we do not connect, and even the problem that they haven’t seen the decision guidance or that the computer crashes*”.

Trainer and observer judged the nurse’s competence as sufficient to conduct a virtual decision coaching. To enhance the nurse’s self-efficacy, a further training session with a patient simulation could be provided and for the first decision coaching sessions a technical support was offered.

## Discussion

The new training concept that focusses on the special needs of the new target group and also includes basic training in communication skills was feasible and well accepted. Role plays conducted with peers were maintained and supplemented with role plays with simulated patients. This combination seems to be a good opportunity to enhance nurses’ empathetic abilities on the one hand and to train frequent issues and needs of women facing these particular decisions on the other hand. This has been previously observed in other shared decision-making-trainings [48].

Our intention was to train specialized nurses. Recent curricula for oncology and breast care nurses aim to impart competences in decision support [49]. However, most nurses do not have substantial competences in decision coaching and evidence-based risk communication as recently claimed by a novel NICE guidance for shared decision-making [50].

Strengthening the nurses’ role to incorporate interventions facilitating shared decision-making such as decision coaching would equally empower affected women and nurses. Single studies have demonstrated the effectiveness and feasibility of nurses delivering such interventions [28, 51, 52].

However, the centers for familial breast and ovarian cancer faced difficulties in recruiting adequate staff for this new role. In Germany, breast care and oncology nurses are hardly available on the open labor market. Thus, the training was adapted to the needs of the (unexpected) heterogeneous target group. Exercises for independent study were created for nurses in preparation of the training and the practical exercises during the training were extended. Considering the implementation of a new working field for specialized nurses, an initial training in the centers for familial breast and ovarian cancer gains crucial importance, especially if the nurses have not absolved a further training in advance.

Study centers designated a few nurses for the role of the study nurse as well as the decision coach in the context of the EDCP-BRCA-project. Due to the special situation of scheduling the kick off meeting of the main study and the training simultaneously, nurses were confused about the aim of the training and their role in the study. We did not expect this situation to recur.

A nurse’s motivation to become a decision coach seems to be an essential factor for a successful training. This has also been shown previously in the process evaluation of the original training and other trainings [28, 29, 48, 51].

Due to the COVID-19 pandemic, we were obliged to convert the decision coaching into a virtual format. Experiences with this format in other entities of decision coaching are rare [21]. However, it seems to be a promising approach that could - irrespective of a pandemic - enhance the reach and acceptability of decision coaching.

One strength of our study was the triangulation of multiple perspectives to test the feasibility and acceptability of the adapted training program. However, there were several limitations. Half of the participants were not part of the originally planned target group. This limited our results. However, as mentioned above, it is challenging to find nurses with this qualification and therefore we adapted the training and supplemented an initial training taking the special needs of this target group into account. According to our experiences with breast care nurses supporting women with ductal carcinoma in situ, we are confident that the training combined with the initial training concept is now adequate for the target group. We were not able to test nurses’ decision coaching skills with real patients in the clinical setting. However, we expect that nurses will be able to adequately provide decision coaching. Furthermore, at the beginning of the main trial, trainers will supervise the nurses’ first two decision coaching consultations. In addition, only one researcher (BBH), who was also involved in the trainings and had experience in qualitative data analysis, conducted the analysis. Nevertheless, the results were discussed with another researcher (BBH, AS) and revision was guided by the discussion of the results with the trainer team.

## Conclusions

Specialized nurses are hardly available and not implemented in the field of familial breast and ovarian cancer in Germany. However, training of nurses with less specialized qualifications seems feasible in the field of familial breast and ovarian cancer. Decision coaching in a virtual format seems a promising approach in rural areas, within a pandemic situation or where the availability of special centers is restricted. Further research is needed to evaluate its feasibility and acceptability as well as the effectiveness. In a next step, the training will be used in preparation of the main trial.

## Supplementary Information

**Additional file 1.** COREQ (COnsolidated criteria for REporting Qualitative research) Checklist

**Additional file 2.** Coding guideline

## Data Availability

The data set will be available to all principal investigators. The datasets generated for the current study will not be publicly available but will be available from the corresponding author on reasonable request.

## Abbreviations

*BRCA1/2* gene: breast cancer susceptibility gene 1/2
COREQ: COnsolidated criteria for REporting Qualitative research
CReDECI: Criteria for Reporting the Development and Evaluation of Complex Interventions in healthcare
EDCP-BRCA: German acronym meaning evaluation of a decision coaching program to support preventive decisions of healthy female BRCA 1/2 gene mutation carriers
