# Supplementary material for: Nurse-led decision coaching by specialized nurses for healthy BRCA1/2 gene mutation carriers - adaptation and pilot testing of a curriculum for nurses: a qualitative study
Source: BMC Nurs. 2022 Feb 10;21:42. doi: 10.1186/s12912-022-00810-8 (PMC8829999; doi:10.1186/s12912-022-00810-8)
Supplement: Supplementary file 2 — Additional file 2. Coding guideline [file 12912_2022_810_MOESM2_ESM.docx]

# Additional file 2: Coding guideline

| **Category** | **Definition** | **Anchor examples** |
| --- | --- | --- |
| **Framework conditions of the training** | **Contextual factors** | „protected environment“ (Participant, after module 2) |
| Organization of the training | Information flow in preparation of the training | „Very detailed, sometimes I was wondering for practical relevance, what is the job in the study, cannot really imagine what it would be. Maybe it’s because they want to establish a new role in the working field.” “Would have liked more insight through role plays. It would have been helpful, to have more practical links at the end” (Participant at the end of module 1) |
| **Interaction** | **Exchange between the involved people** |  |
| Role of teachers | Appropriateness of performance | “*All trainers were clear about their role and work packages during the training*” (Observer module 1, day 1) |
| Relationship between lecture and work phases | Balance between lecture and work phases | “*Varied methods, vivid*.” (Participant, module 1, day 2) |
| Interaction between learners | Mutual interaction of learners regarding their actions and communication | Statement of a participant: “*We are all so open here, so let me tell you something*...” (Participant module 1, day 2) |
| Interaction between learners and teachers | Mutual interaction of learners and teachers regarding their actions and communication | „*Very committed participants, good exchange between each other and with the trainers*” (Observer, module 1, day 3) |
| **Schedule** | **Smooth running of teaching/adequacy of the training programme** |  |
| Adequacy of the time frame (timing and duration of the training) | Adequacy of the time frame (timing and duration of the training) | „*Overall, every participant had only 10 minutes training with the simulated patient (not enough)*” (Trainer, module 2, day 1) |
| Common thread | Structuring of the lessons, transparency of the teaching process | *„I as a non-expert observer missed a brief description of the nurses’ task profile as a decision coach and of the required skills (e. g. expertise in terms of gene mutation, test accuracy, communication skills etc.) Furthermore, no general learning goals for the entire training were derived.”* (Observer, module 1, day 1) |
| **Transparency of teaching and learning goals** | **Clarity/transparency of teaching and learning goals for the learners** | “*Presumably, theoretical basics are important. Trials and so on are important (even its appraisal), what could this look like. Everyone wondered if they would be able to work in it, would they be able to cope with it.*” (Feedback participant, at the end of module 1)” |
| **Content** | **Comprehensibility, acceptance and adequacy of contents** |  |
| Reflection on contents | Reflection on contents in terms of the gathered knowledge | “*Discussion: A controversy arises in terms of the self-examination of the breast: the decision aid is inconsistent* “ (Trainer, module 1, day 3) |
| Comprehensibility | Comprehensibility of the contents of the training | „*The training was experienced as being demanding but interesting*” (Observer, module 1, day 3) |
| Comprehensiveness of contents | Sufficiency of contents or lack of input, missing examples that contribute to comprehension | “*Wish of a participant for more information about ‘how people make decisions’*” (Trainer, module 2, day 1) |
| Acceptance of the contents | To what extent can the participants accept the contents of the training? | „*Demanding, totally interesting, so broadly positioned, interestingly presented, probably this is due to you*” (Participant at the end of module 1) |
| Target group adequacy | How do the provided contents match the needs of the target group? | “*The initial confusion and frustration declined with module two, as it made sense for all training contents*” (Participant module 2, day 1) |
| **Methods** | **Use of teaching methods** |  |
| Acceptance of the teaching methods | Acceptance of the teaching methods by the learners | „*doesn´t like role plays*” (Participant after module 2) |
| Realisation of the teaching methods | Successful implementation of the teaching methods (e.g. group work) | „*Introduction into the decision guidance: A few participants made notes on it, others did not.*” (Trainer, module 2, day 1) |
| **Materials** | **Use of materials** |  |
| Design of teaching materials | Comprehensibility, design and logical structure of the learning and teaching materials | Training folder: “*Sometimes the participants have to turn the pages frequently, a few of them skipped ahead*” (Trainer, module 1, day 2) |
| Design of coaching materials | Comprehensibility, design and logical structure of the coaching materials | „*The active exercises with the simulated patient showed that the participants were not familiar with the materials (decision aid and decision guidance)*” (Observer, module 2, day 1) |
| Confidence in working with coaching materials | Handling of the and familiarity with coaching materials | *Feedback: “You did not feel confident”* (Participant module 2, day 1) |
| **Practical relevance and feasibility** | **Applicability in practice** | “*The content of the test accuracy led to clarifying questions, or were queried in general (practical relevance and relation to the study?*)” (Observer, module 1, day 3) |
